# New Insights Into the Physiopathology of COVID-19: SARS-CoV-2-Associated Gastrointestinal Illness

**DOI:** 10.3389/fmed.2021.640073

**Published:** 2021-02-18

**Authors:** Christian A. Devaux, Jean-Christophe Lagier, Didier Raoult

**Affiliations:** ^1^Aix-Marseille University, IRD, APHM, MEPHI, IHU-Méditerranée Infection, Marseille, France; ^2^CNRS, Marseille, France

**Keywords:** COVID-19, SARS-CoV-2, gastrointestinal illness, microbiota, butyrate, tryptophan, vitamin D

## Abstract

Although SARS-CoV-2 is considered a lung-tropic virus that infects the respiratory tract through binding to the ACE2 cell-surface molecules present on alveolar lungs epithelial cells, gastrointestinal symptoms have been frequently reported in COVID-19 patients. What can be considered an apparent paradox is that these symptoms (e.g., diarrhea), sometimes precede the development of respiratory tract illness as if the breathing apparatus was not its first target during viral dissemination. Recently, evidence was reported that the gut is an active site of replication for SARS-CoV-2. This replication mainly occurs in mature enterocytes expressing the ACE2 viral receptor and TMPRSS4 protease. In this review we question how SARS-CoV-2 can cause intestinal disturbances, whether there are pneumocyte-tropic, enterocyte-tropic and/or dual tropic strains of SARS-CoV-2. We examine two major models: first, that of a virus directly causing damage locally (e.g., by inducing apoptosis of infected enterocytes); secondly, that of indirect effect of the virus (e.g., by inducing changes in the composition of the gut microbiota followed by the induction of an inflammatory process), and suggest that both situations probably occur simultaneously in COVID-19 patients. We eventually discuss the consequences of the virus replication in brush border of intestine on long-distance damages affecting other tissues/organs, particularly lungs.

## Introduction

**Figure 1:**
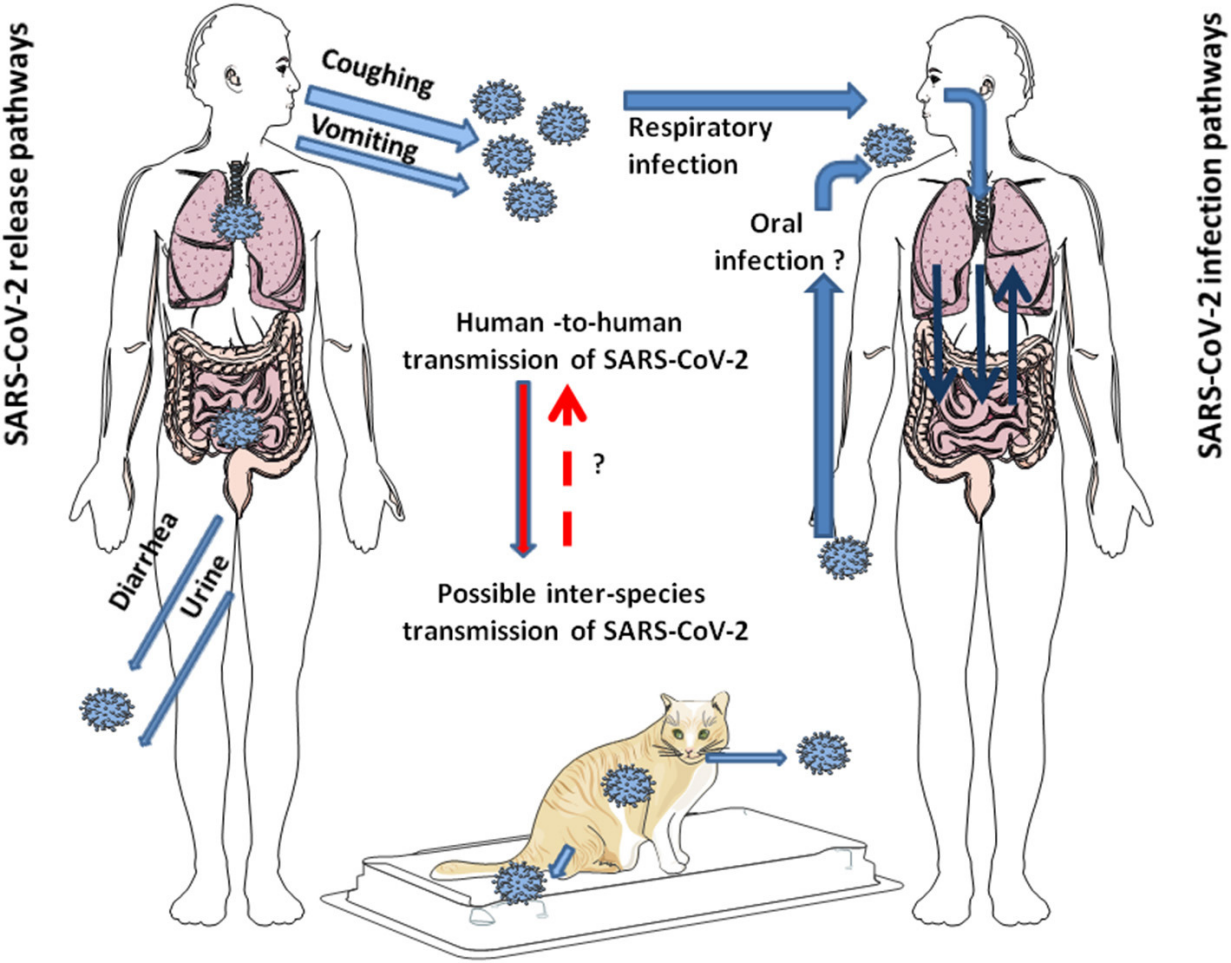
Schematic representation of the modes of transmission of SARS-CoV-2 among humans. SARS-CoV-2 is released from infected individuals by coughing, vomiting, and through diarrhea and urine. Airborne is considered the primary mode of human-to-human transmission of SARS-CoV-2. After infection of the respiratory tract, the virus can leave the lungs via circulation to induce secondary foci in other organs, including GIT. Whether SARS-CoV-2 can directly infect the GIT by the oral route in humans seems possible since it is stable at acidic pH, but it is still under debate. Additionally, it has been shown that SARS-CoV-2 can jump back and forth between humans and animals. This has been largely reported with minks. In pets, such as cats, human-to-cat transmission of SARS-CoV-2 and cat-to-cat respiratory transmission of SARS-CoV-2 was demonstrated, not yet for cat-to-human transmission of SARS-CoV-2.

One year after the first outbreak of **Co**rona**vi**rus **d**isease 20**19** (COVID-19) in China, the disease has emerged as a world pandemic with fatality rate around 2.27%, causing more than 1.57 million deaths for 68.95 million people infected worldwide on 10 December, 2020 (https://coronavirus.jhu.edu/map.html). Although its etiological agent, SARS-CoV-2, is mainly a lung-tropic virus, it is responsible for multi-organ failure in patients with severe forms of the disease (1, 2). To enter susceptible cells, this virus binds to the angiotensin I converting enzyme 2 (ACE2) (3). Some of the harmful effects of SARS-CoV-2 infection are associated with the dysregulation of the renin angiotensin system (RAS) pathway and thrombosis since the virus receptor, the ACE2 monocarboxypeptidase, acts as a regulator of blood pressure homeostasis through its ability to catalyze the proteolysis of Angiotensin II (AngII) into Angiotensin (1, 3–8). Yet, many papers reported clinical dysfunction with various extra-pulmonary symptoms that are likely RAS-independent, in particular intestinal disorders (5–8). SARS-CoV-2 induces diarrhea, nausea abdominal pain and vomiting as onset symptoms in patients with COVID-19 (5, 9). Zhang and collaborators reported that 8.0–12.9% of COVID-19 patients suffered from diarrhea (10). Indeed, gastrointestinal tract (GIT) symptoms were observed in 5–80% of COVID-19 patients depending on the cohort studied, and these symptoms sometimes precede the development of respiratory tract symptoms (6–8, 11–13). Digestive symptoms, in particular diarrhea, have been reported as symptoms associated with a mild form of the disease (without difficulty to breath and without low blood oxygen levels), and people with GIT-symptoms were much more likely to have the SARS-CoV-2 detected in their stool samples (14). The process by which SARS-CoV-2 reaches the intestine is not yet clear, and could occur either by the bloodstream (with or without a hepatic stage) or by the oral-intestinal route (from the trachea to the esophagus and intestine) (Figure 1). If the correlation between mild GIT-symptoms and SARS-CoV-2 detection in stool was confirmed in some studies, other reports suggest that COVID-19 patients with GIT-symptoms might have a more severe form of the disease including the development of severe respiratory disorders (15, 16). Moreover, it was reported that SARS-CoV-2 can be detected in anal swabs and stool samples in almost 50% of COVID-19 patients (17, 18) and that duration of SARS-CoV-2 shedding from stool was longer than that from respiratory samples (19). This suggests that the gut is an active site for SARS-CoV-2 replication. Intestinal biopsies of COVID-19 patients have allowed to evidence the presence of replicating SARS-CoV-2 in epithelial cells of the small and large intestine (20), highlighting an appropriate combination between the virus spike sequence, the expression of ACE2 and host protease required for spike processing during viral entry (21). Each day, additional SARS-CoV-2 genomes are sequenced. Yet, there is a massive knowledge gap regarding the SARS-CoV-2 clade(s) that establish productive infection in enterocytes. It was estimated that levels of SARS-CoV-2 RNA in stools can range from 5.5 × 10^2^ to 1.2 × 10^5^ copies /mL, still much lower than in nasopharyngeal fluids where SARS-CoV-2 RNA ranges from 10^5^ to 10^11^ copies/mL (22, 23). However, there are studies reporting fecal shedding of 1.0 × 10^7^ copies/mL (17, 24). Substantial amounts of SARS-CoV-2 viral RNA can be detected in the stool by polymerase chain reaction even after the patients' respiratory samples tested negative for the virus (25–27). Live virus can be detected by electron microscopy in SARS-CoV-2 positive fecal specimens (28), however virus isolation from feces remains difficult (17, 29). This does not allow to exclude the risk of possible fecal-oral transmission (30–34). Although SARS-CoV-2 has been found extremely stable in a wide range of pH values (pH 3–10) (35), it is possible that SARS-CoV-2 may be inactivated in stool samples due to bioactive molecules present in stimulated low pH human colonic fluids (36). It might include mitochondrial antiviral signaling protein (MAVS)-mediated type III interferon (IFN) induction, such intestinal antiviral innate immunity rendering the viral culture more difficult to establish (37, 38). Indeed, it was recently reported that SARS-CoV-2 infection of enterocytes is associated with an extremely robust innate immune response mediated by type III interferon, which inhibits SARS-CoV-2 replication and *de novo* production of the virus (39). This also questions the nature of the molecular cross-talk set-up between SARS-CoV-2, cells from the intestinal barrier, immune cells present in this tissue and the gut microbiota (40–43).

## Lessons to be Learned from Other Coronaviruses to Fill the Knowledge Gap on GIT Specific SARS-CoV-2

Coronaviruses are among the most common pathogens identified in the feces of mammals, such as cats and bats (44–46). Several animal coronaviruses are natural enteric pathogens, they cause GIT diseases, and spread by the fecal-oral route, such as the polytropic strains of murine betacoronavirus **M**ouse **h**epatitis **v**irus (MHV) that uses the carcinoembryonic antigen molecule CEACAM-1 as receptor and causes disease in housed rodent colonies (47). The MHV-1 induces severe pneumonitis, while several strains (e.g., MHV-D or MHV-Y) were found enterotropic. The coronavirus S glycoprotein has a major influence on MHV viral tropism (48). Swine **T**ransmissible **G**astro**e**nteritis corona**v**irus (TGEV) and **S**wine **A**cute **D**iarrhea **S**yndrome coronavirus (SADS-CoV), **C**anine alphacoronavirus (CCoV), **B**ovine betacoronavirus (**B**CoV), and avian gammacoronaviruses including **T**urkey coronavirus (TCoV), **Q**uail coronavirus (Q CoV) and **G**uinea**f**owl coronavirus (GfCoV), are associated with GIT disease (49, 50). The intestinal form of pig TGEV that infects piglets, has been replaced worldwide by a much less pathogenic **P**orcine **r**espiratory **c**oronavirus (PRCV) pneumotropic strain that differs from TGEV by a few genomic deletions including a 672 nucleotides deletion in the 5′ region of the spike (51). With the **F**eline coronaviruses (FCoV), some isolates are defined as low-virulence **F**eline **e**nteric **c**orona**v**irus (FECV) whereas others are defined as highly virulent **F**eline **i**nfectious **p**eritonitis **v**irus (FIPV) and the ability to infect macrophages is an essential virulence factor; the FIPV spike protein was found to be the determinant for efficient macrophages infection (52). In addition, traces of the genome of almost all the coronaviruses circulating in the human species HCoV-OC43, -HKU1,−229E, -NL63, SARS-CoV, and MERS-CoV have been found in stool of infected humans (53–57). Moreover, 30% of patients with Middle East respiratory syndrome (MERS) and 10.6% of patients with SARS-CoV-1 presented diarrhea (43). Although it was previously reported that no live SARS-CoV-1 could be detected in stool samples from SARS patients despite detection of SARS-CoV-1 mRNA (55), it was also hypothesized that MERS-CoV and SARS-CoV-1 could be transmitted through the fecal-oral route (58, 59). During the episode of SARS-CoV-1 in Hong Kong in March 2003, a study investigating the possible origin of the outbreak suggested that the contamination occurred through bathroom floor drains with dried-up U-traps at the Metropole Hotel in Kowloon, which was a passageway through which residents came into contact with small droplets containing viruses from the contaminated sewage after the stay of a Guangzhou professor who had been caring for patients with atypical pneumonia (60).

Coronavirus strains within a host usually represent mixtures of different viral populations illustrating an adaptation to the host with selection pressure working on a quasispecies basis. The viral mutation rate for RNA viruses was estimated from 10^−6^ to 10^−4^ misincorporation per nucleotide (61, 62). Many evidence support that RNA viruses exist as quasispecies and are characterized by continuous genetic variation within populations which is the result of high error rates of RNA-dependent RNA polymerases. Mounting evidence indicates that over time, quasispecies development may promote the emergence of new viral species with a tropism perhaps distinct from that of the first viral isolates. With TGEV, mutations of two nucleotides (nt) at positions 214 and 655 in the spike induced a shift in tropism from enteric to respiratory tropism (63). Similar observations of intra-host quasispecies were reported with BCoV that split between enteric and respiratory variants with the AH65-R BCoV and AH187-E BCoV being able to change their tropism after multiple passages in tissue culture (64). With FIPV, genetic variation and recombination were reported within the same cat and between cats (65, 66). A single nucleotide change within the S gene encoding the fusion peptide was found in 96% of FIPVs from cats with the wet and dry form of FIPV, but was absent from FECV (67). Another study reported mutations in the region of the S1/S2 cleavage site of FIPVs affecting the efficiency of cleavage of the spike protein by furin (68). Quasispecies were observed in the 5′ untranslated regions of the pig CoV HKU15 and also in four positions with 2 nt substitutions and two indels (69). Genetically diverse populations of SARS-like CoV are present in geographically closely related Chinese horseshoes bats (70). It was reported that SARS-CoV-1 exists as a quasispecies in individual patients with nine recurrent non-synonymous variant sites in the spike (71). The presence of MERS-CoV-associated coronavirus quasispecies was also reported (72).

## Tissue-Specific Patterns of SARS-CoV-2 Variants

In the Syrian hamster model of COVID-19, the SARS-CoV-2 was found to replicate in the animal lungs and to induce severe lung lesions similar to commonly reported lung damages in humans (73, 74). Beside the hamster lungs, among non-respiratory tract tissues only the intestinal tissues demonstrated viral antigen expression in association with severe epithelial cell necrosis, intestinal villi damage and increased *lamina propria* mononuclear or neutrophilic cell infiltration. Next generation sequencing (NGS) was used to study SARS-CoV-2 intra-host variability and identify possible tissue-specific patterns and signature of variant selection for upper (URT) and lower respiratory tract (LRT) from six COVID-19 patients (75). The presence of quasispecies was observed in this study with differences between the URT and LRT variants indicating a quasispecies compartmentalization. Yet, no significant nucleotide differences (signature) were detected between URT and LRT variants in the S glycoprotein. Similar results have been reported with characterization of quasispecies differences between anatomical sites (URT vs. LRT), but also from one day to the next with sequential samples from a single patient, suggesting a complex dynamic distribution of variants (76). It has been recently reported that SARS-CoV-2 can be detected in multiple organs including pharynx, liver, pancreas, kidneys, heart and brain (77, 78). To the best of our knowledge, no study has been performed so far to identify the possible signature of SARS-CoV-2 variant selection for respiratory vs. intestinal tropic viruses, such work is currently under way in our institute.

It is currently well-established that intrahost SARS-CoV-2 variability is frequent across the viral genome in COVID-19 patients (79–81). It was also reported that intrahost SARS-CoV-2 variability is higher in cancer patients compared to non-cancer counterparts (82). Several non-synonymous mutations have been reported in the spike of SARS-CoV-2. Interestingly, it was reported that SARS-CoV-2 accumulates deletions very close to the S1/S2 cleavage (RRAR∧S) and mutations that can affect the furin cleavage site (83–85). By similarity with other coronaviruses, it is likely that strains of SARS-CoV-2 exhibiting a specific tropism for GIT will soon be identified. Finally, the hypothesis that SARS-CoV-2 could be transmitted through the fecal-oral route remain the subject of intensive research (86).

## Tissue Distribution of the ACE2 Viral Receptor and SARS-CoV-2 Viral Tropism

The viral receptor, ACE2, is a 805 amino acids type I cell-surface glycoprotein distributed broadly on type I and type II alveolar epithelial cells (87), in the arterial and venous endothelial cells and the arterial smooth muscle (88), and is also expressed in the renal, the cardiovascular and gastrointestinal tissues (89). ACE2 was also reported on the epithelial cells of the oral mucosa (90). Using a Syrian Hamster animal model of SARS-CoV-2 infection, it was recently reported that oral inoculation of SARS-CoV-2 established mild pneumonia in 67% of animals exposed to the virus and caused intestinal inflammation (91). The expression of ACE2 on enterocytes of the small intestine was reported by Hamming et al. (88), with the highest expression found in the brush border of intestinal enterocytes (92, 93), the main role of which is to ensure the absorption of nutrients. According to a preprint (not peer reviewed) by Wang et al., ACE2 is highly expressed on colonocytes, slightly expressed on colonocytes-bestrophin (BEST4) anion channel positive, very slightly expressed on enteroendocrines cells and Paneth cells, almost undetectable in goblet cells, and tuft cells (94). Colonocytes were also found at single cell resolution to overexpress genes regulating viral entry, budding, and release (including the chromatin modifying proteins CHMP1A, CHMP1B, CHMP2A, CHMP2B, CHMP3, CHMP4B, CHMP4C, that are members of the endosomal sorting complex required for transport ESCRT family; the vacuolar protein sorting associated proteins VPS4B, VPS28, VPS37B; the programmed cell death six interacting protein PDCD6IP and the multivesicular body subunit MVB12A, that function within the ESCRT pathway; the vesicle-associated membrane protein-associated protein VAPA involved in membrane trafficking; the poliovirus receptor related PVRL2, a component of tight junctions; and the cadherin CDH1/E-cadherin that maintain epithelial tight junctions).

ACE2 also suppresses intestinal inflammation by maintaining amino acid homeostasis (95, 96). SARS-CoV-2 was found to infect human small intestinal organoids established from primary gut epithelial stem cells and proliferative progenitor or Apolipoprotein A1^+^ enterocytes (97). This is likely how SARS-CoV-2 mediates the invasion of the GIT and its local amplification. Yet, beside ACE2, the molecules involved in SARS-CoV-2 early stages of infection may differ. In pneumocytes, it has been well-established that following ACE2 receptor engagement SARS-CoV-2 is processed by a type II transmembrane serine protease, TMPRSS2 prior to membrane fusion. Although both ACE2 and TMPRSS2 are highly expressed in the GIT, it was reported that these molecules are not co-expressed on enterocyte, TMPRSS2 being expressed on ACE2^neg^ intestinal epithelial cells and not mature enterocytes; yet, for the processing of the viral spike (S), TMPRSS2 can probably be replaced by other serine proteases of the same family, such as TMPRSS4, highly expressed in ACE2^+^ mature enterocytes (36). It was previously reported with SARS-CoV-1 that the sheddases ADAM17 and ADAM10 can cleave ACE2 but only the cleavage by TMPRSS2 resulted in augmented SARS-CoV-1 spike driven entry (98, 99).

## The Function of ACE2 in the Gastrointestinal Tract

Once dietary proteins have been hydrolyzed by the action of proteases and by brush-border membrane-bound peptidases, trans-epithelial absorption of amino acids across enterocytes involves amino acid transporters (100). ACE2 can cleave carboxy-terminal amino acids from nutrients proteins/peptides and its proteolytic activity has a pH optimum of 6.5 (90% efficiency at pH 6.0–7.5), compatible with the intestinal pH that ranges from 7.3 to 7.7 (101, 102). ACE2 is also required for expression of the sodium-dependent neutral amino acid transporter B^0^AT1 and amino acid (proline) SIT1 transporters on the luminal surface of intestine epithelial cells and the two transporters co-localize with ACE2 along the brush-border membrane of duodenum and terminal ileum enterocytes on villi (103, 104). In ACE2 deficient mice, B^0^AT1 is absent from the small intestine (103). Expression of the B^0^AT1 gene is controlled by the activation transcription factors HNF1α and HNF4α (105). A close association of B^0^AT1, ACE2, and aminopeptidase N (APN) in the brush-border membrane was reported (102). Fairweather et al. suggested that B^0^AT1 trafficking and expression in the apical membrane of enterocytes is largely dependent on ACE2, whereas optimal functioning to changing dietary conditions requires association with APN. It was reported that ACE2 regulates the gut homeostasis, the expression of antimicrobial peptides and the gut microbiota (95, 96). According to Hashimoto et al. (95) *ACE2* knock-out (KO) mice had reduced levels of neutral amino acids in the serum, displayed impaired tryptophan uptake, and showed an altered composition of the microbiota (likely a loss of bacteria sensitive to oxidative stress), which could be restored by tryptophan administration. Tryptophan enhances expression of tight junction proteins Claudin-3, Claudin-4, and Zonula Occludens ZO-1 and ZO-2 (106). When *ACE2* knock-out (KO) mice were challenged with dextran sodium sulfate a profound inflammatory reaction was observed (107). Fecal transplantation of this microbiota into germ-free animals trigger infiltration of inflammatory cells and an increased propensity to develop severe colitis. An antibiotic treatment rescued bloody diarrhea in the ACE2 deficient mice colitis model. This influence of ACE2 on the gut microbiota composition was confirmed in another study (108). The ACE2 regulation of gut homeostasis was RAS-independent and ACE2 regulate the innate immunity. This possibly explains the diarrhea sometimes observed with SARS-CoV-2 patients, and support the use of antibiotic treatment in COVID-19 patients.

Epithelial cells, such as epithelial enterocytes, goblets cells, Paneth cells, and intestinal stem cells express the nucleotide-binding oligomerization domain 2 (NOD-2) which sense the bacterial muramyl dipeptide (MDP), attracts receptor-interacting serine/threonine kinase 2 (RIP2), transforming growth factor β-activated kinase 1 (TAK1) and TAK1 binding proteins 2 (TAB2) or TAB3. This complex also induces the activation of both MAP kinase (MAPK) and NF-κB which contribute to activate the secretion of antimicrobial peptides Reg3γ, α-defensin, such as HD5 and HD6, β-defensin, and lysozyme (109). It was reported that human defensin-5 (HD5), the most abundant α-defensin (a lectin-like peptide able to bind lipids and glycosylated proteins), secreted by intestinal Paneth cells, interacts with ACE2 at an affinity of 76.2 nM (110). In the ileal fluid the HD5 is present in abundance (6–30 μg/mL; around 2–8 μM) so that HD5 can compete with SARS-CoV-2 for binding to ACE2 α-helix 1 and loop 2. Wang et al. have found that adding HD5 to Caco2 cells significantly reduced SARS-CoV-2 infection (111). Interestingly, α-defensins have been linked to atherosclerosis being involved in the lipoprotein metabolism in the vessel wall and favoring LDL and lipoprotein (112, 113).

## GIT Disease: The Direct SARS-CoV-2 Effect Model

ACE2 has been shown to have a potent interaction with SARS-CoV-2 S glycoprotein with an affinity of 14.7 nM which is about 10- to 20-fold higher than that of ACE2 binding to SARS-CoV-1 S protein (114). It was reported that ACE2 is predominantly expressed in CD26^+^Epcam^+^ CD44^−^CD45^−^ mature enterocytes of the gut epithelium and present in both duodenum and ileum (103). It was documented that the membrane-bound TMPRSS2 can cleave ACE2 as well as the viral spike thereby promoting SARS-CoV-2 entry into the target cells. TMPRSS2 is expressed on ACE2^−^ intestinal epithelial cells while two other serine proteases in the same family, TMPRSS4 and ST14/matriptase are highly expressed in ACE2^+^ mature enterocytes and, TMPRSS4 was found to increase SARS-CoV-2 infectivity (36). SARS-CoV-2 induces syncytia formation between intestinal epithelial cells (36). This process is likely to lead to subsequent cytopathic effect and local damages that could explain the GIT symptoms observed in COVID-19 patients. In addition, the SARS-CoV-2 infection is likely to trigger innate immunity by the activation of pattern recognition receptors (PRRs) able to recognize components termed pattern associated with molecular patterns (PAMPs), including viral antigens and both cellular stress signals and damaged tissue. PAMPs are recognized by the amino-terminal leucine -rich repeat of toll-like receptors (TLR) type I transmembrane proteins expressed at the cell surface or in endosomes. TLR are classified into six major families and include TLR-3 which recognizes double stranded RNA (dsRNA), TLR-7, and TLR-8 which detects single-stranded RNA (ssRNA) while TLR-9 engages unmethylated CpGDNA (115). When activated these receptors expressed in the intracellular endosomes, trigger signals (e.g., MyD88 or TIR-domain-containing adaptor) inducing interferons (IFNs). This is expected to decrease viral spread by establishing an antiviral state in uninfected neighboring cells. The TLR-3 receptor is expressed on endosomes of mature gut epithelial cells whereas TLR-4 is expressed only in crypt epithelial cells and its expression is lost as the cells mature and move toward the gut lumen (116).

Previous studies conducted on SARS-CoV-1, revealed that no modulation of TLR was observed in monocytes but the infection was associated with over-expression of chemokine receptors CCR-1, CCR-3, and CCR-5 and TNF-related apoptosis inducing ligand (TRAIL) which may induce lymphocytes apoptosis and lymphopenia (117). TLR-3 agonist poly(I:C) and TLR-4 agonist lipopolysaccharide (LPS) were found to be protective against SARS-CoV-1 and MERS-CoV infection in mice (118, 119). In addition, in a model of TLR-3/TLR-4-deficient mice, these mice were found to be more susceptible to SARS-CoV-1 infection than wild type animals (120). Regarding SARS-CoV-2, it is worth noting that lymphocytes count has been found to be a marker of the severity of COVID-19, lymphopenia on admission being associated with poor outcome of the disease (121, 122). Interestingly, in SARS-CoV-2 infection, at least 3.5% of patients with severe COVID-19 have mutations in IFN genes affecting antiviral defense and 10% of patients produce auto-antibodies against type I IFN suppressing immune response (123, 124).

The nucleotide-binding oligomerization domain 2 (NOD2), a recognition receptor that senses MDP bacterial peptidoglycan-conserved motifs in cytosol is expressed in Paneth cells. After its engagement with MDP, NOD2 triggers the production of host defense peptides (HDPs; previously named AMPs for antimicrobial peptides) as well as cytokines and chemokines stimulating the immune response from both epithelial and immune cells (125, 126). It was recently reported that the α-defensin HD5 (but not HD6) present at the level of intestinal mucosa can bind ACE2 at high affinity (39.3 nM) thereby inhibiting the interaction between the S glycoprotein of SARS-CoV-2 and ACE2 in a dose-dependent manner (110). It could be of importance for SARS-CoV-2 during its colonization of the intestinal epithelium to down-regulate HD5 and this could be achieved by acting on the neutral amino acid transporter B^0^AT1. In addition, immunoglobulin A (IgA are produced by B lymphocytes localized in the intestinal lamina propria) acts as host defense against viruses. Recently different studies reported that the serum level of SARS-CoV-2 specific IgA is positively correlated with COVID-19 severity (127, 128). This suggested a massive specific immune response activation against SARS-CoV-2 part of which could come from the synthesis of IgA by B lymphocytes present in the intestinal mucosa. It was previously reported that the intestinal IgA are recruited during inflammatory processes (129).

Very recently it was reported that the enhanced human spreading of SARS-CoV-2 compared to SARS-CoV-1 could possibly be explained by the presence of a polybasic furin type cleavage site, RRAR∧S, at the S1/S2 junction in the SARS-CoV-2 spike which is not found in SARS-CoV-1 and likely primes the fusion activity and could potentially create additional cell surface receptor binding sites. Under such condition, neuropilin-1 (NRP-1) known to bind furin-cleaved substrates could be an entry cofactor that potentiates SARS-CoV-2 infectivity (130). NRP-1 and its related NRP-2 transmembrane protein are 120–130 kDa multifunctional non-tyrosine kinase receptor known to interact with both the class 3 semaphorins and heparin-binding members of the vascular endothelial growth factor (VEGF) family, as well as other growth factors in epithelial cells (131, 132). Both NRP-1 and NRP-2 are expressed in the GIT (133, 134). NRP-2 was initially found expressed at the basolateral side of the serotonin-producing enteroendocrine cells in small intestine (133) but both NRP-1 and NPR-2 were later found to co-localize with cells that express chromogranin-A (CgA), a general marker of enteroendocrine cells (135). The presence of NRP-1 in the intestine (about 10% of CgA^+^ cells express NRP-1 and GPR41/GPR43), could therefore increase the intestinal infectivity of SARS-CoV-2. In the crypts of colonic epithelium these cells express VEGF in their granules, suggesting that VEGF may have a role in the maintenance and control of the permeability of the capillary system (136).

**Figure 2:**
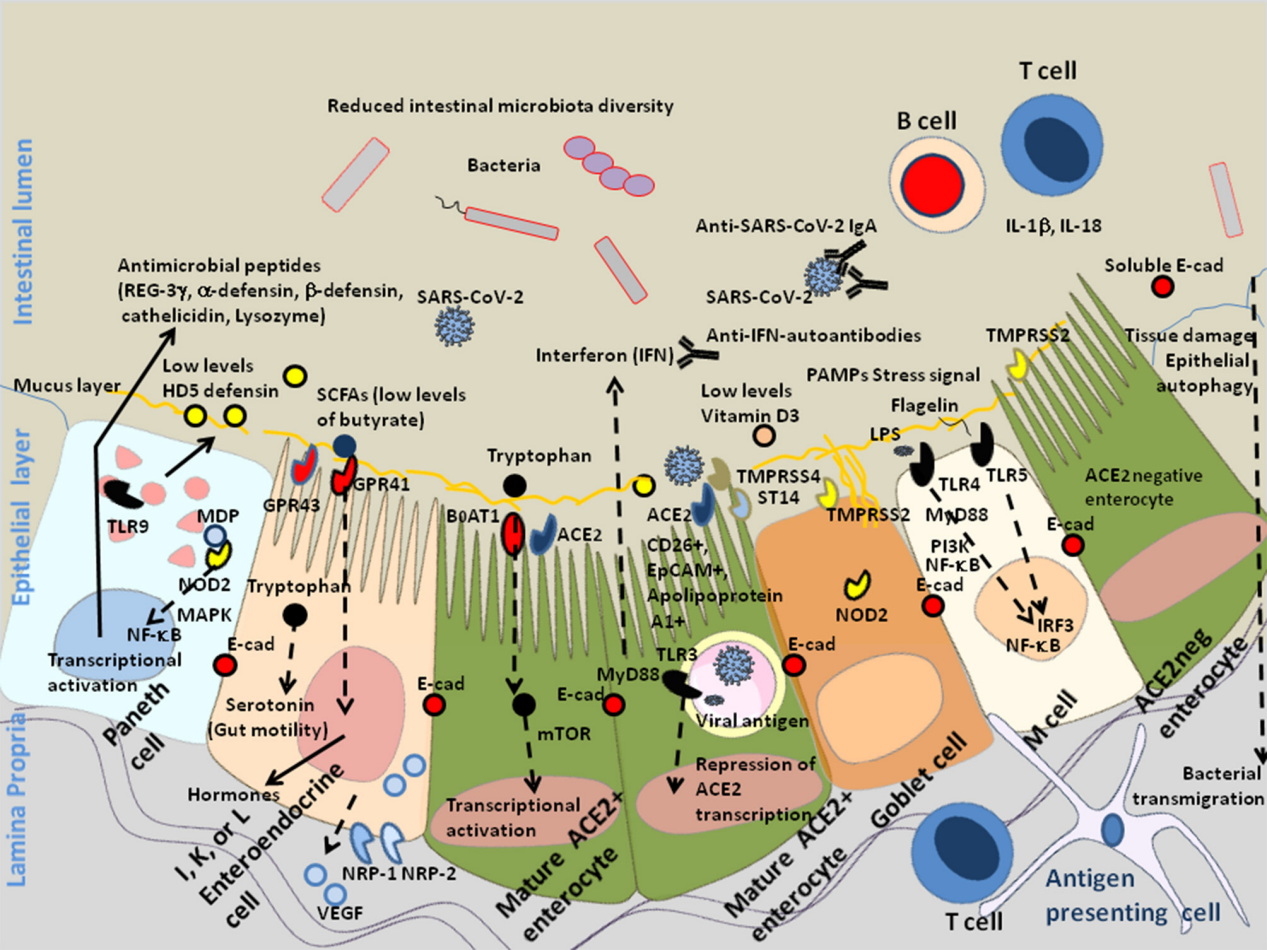
Schematic diagram of SARS-CoV-2 infection of mature enterocytes and consequences on intestinal dysbiosis. Different cell types (enterocytes, Paneth cells, globet cells, M, cells, enteroendocrines cells, tuft cells) interact together through tight junctions (homotypic interactions of E-cad in trans) to form a continuous epithelial barrier isolating the luminal content from the internal tissues. The goblet cells secrete the mucins (e.g., MUC2–mucin gel) in the hope of protecting the intestinal epithelium by reducing bacteria attachment while allowing nutrients to be processed by enterocytes. The Paneth cells produce antimicrobial proteins, in response to infection. The enteroendocrines epithelial cells express the FFA2/GPR43 and FFA3/GPR4 surface receptors that bind the SCFAs and trigger signal leading to the regulation of the glucose homeostasis and the secretion of hormones influencing appetite; these cells also express the NRP-1 receptor and produce VEGF. The main role of enterocytes is to ensure absorption of nutriments. Uptake of tryptophan depends on B^0^AT1. ACE2, expressed by enterocytes is necessary for the surface expression of the amino acid transporter B^0^AT1 in the intestinal epithelium. ACE2 mRNA expression is strongly reduced in cells infected by SARS-CoV-2. In the microenvironment where the infection occurs, the villous microfold cells (M cells) expressing the toll-like receptors/TLR detect stress signals, tissue damages, and PAMPs (e.g., lipopolysaccharide /LPS from Gram negative bacteria recognized by TLR-4 and its co-receptor CD14, flagellin, peptidoglycan, lipoproteins, and unique bacterial nucleic acid structures), have a pivotal role in antigen presentation, they uptake antigens from the luminal content, transport (transcytosis and microvesicle uptake), these antigens to their basolateral membrane where they are delivered to the underlying immune cells of the gut-associated lymphoid tissues (GALT), including KLRG1+ dendritic cells and monocytes/macrophages, CD103+ T-cells, KLRG1+ T-cells, and other immune cell subpopulations, which colonize the lamina propria. After their priming, the immune response cells migrate to the site of infection to counteract the pathogen invasion. The doublecortin like kinase 1 (DCLK1+) tuft cells also contribute to innate immunity (i.e., they recognize protozoan and helminth antigens) and produce IL-25 that activate innate lymphoid cells (ILC2) at inducing IL-13 production. It is likely that a decrease in butyrate (protective function) is a consequence of SARS-CoV-2-associated reduced diversity of microbiota. In addition, COVID-19 patients produce less HD5, thereby reducing the overall antibacterial defense. Due to the dysbiosis, stress signals, tissues damages, and recognition of bacterial peptidoglycan-conserved motifs, muramyl dipeptide, and MDP sensed by the nucleotide-binding oligomerization 2 (NOD2) are likely to regulate the production of cytokines IL-1β and IL-18 aimed at restricting bacterial replication and to provoke pro-inflammatory reactions [see review (137) for details]. The activation of cellular and bacterial sheddases reduces the epithelium surface expression of E-cad at the site of infection, resulting in the destruction of adherent's junctions and allowing pathogens' transmigration. We previously speculated that the induction of E-cad on subpopulation of immune response cells (E-cad+ T-cells and CD16+/E-cad+ monocytes) redirects those cells far from the infection site. The release of sE-cad might also serve as a decoy for diverting immune cells from their function through the interaction with E-cad, CD103, or KLRG1 at the surface of immune cells. In addition, SARS-CoV-2 infection is associated with massive production of SARS-CoV-2 specific IgA and lymphopenia. In some patients there are also IFN-specific auto-antibodies that reduce antiviral defense.

Altogether these results indicate that ACE2, TMPRSS4, and NRP-1 are present in the GIT, thus facilitating SARS-CoV-2 infectivity. They also suggest that SARS-CoV-2 reduces the production of HD5 which could otherwise act as a competitive inhibitor for binding to ACE2, induces lymphopenia, and quite frequently (10% of patients) stimulates the production of auto-antibodies against type I IFN, thereby suppressing the antiviral immune response (Figure 2). In those patients the shedding of infectious SARS-CoV-2 into feces could be increased.

## GIT Disease: The Indirect SARS-CoV-2 Effect Model (The Role of Microbiota)

While intestinal symptoms associated with SARS-CoV-2 infection may be due to direct infection of the intestinal epithelium, they may also be due to decreased antibacterial defenses, decreased microbiota diversity, increased intestinal barrier permeability, bacterial translocation and/or systemic leak of endotoxin. *Bacteroidetes* and *Firmicutes* are considered predominant in the gut while *Proteobacteria* are the most abundant in the lung (138, 139). It is usually admitted that the intestinal microbiota can be influenced by respiratory virus infection leading to the development of the disease through the gut-lung axis and that compounds, such as endotoxins, microbial metabolites, and/or cytokines, can travel into the bloodstream connecting both sides of this axis [(10, 140–142). Several recent reports confirm that SARS-CoV-2 replication in the gut is associated with modulation in the diversity of bacterial species present in the GIT, likely reducing host antiviral immune response and aggravating lung damage observed during these infections (143, 144). A study conducted by Gu et al. (145) indicated that, compared to healthy controls, COVID-19 had significantly reduced bacterial diversity and higher relative abundance of opportunistic pathogens, such as *Streptococcus, Rothia, Veillonella*, and *Actinomyces*, which can aggravate the inflammation or be associated with secondary bacterial lung infection. Another investigation (146), confirmed the dysbiosis and reported that a decreased abundance of *Faecalibacterium prausnitzii* (usually one of the most abundant *Firmicutes* in the gut) and an increased abundance of *Coprobacillus, Clostridium ramosum, Clostridium hathewayi, Actinomyces viscosus, Bacteroides nordii* correlated with COVID-19 severity. In addition the abundance in bacterial species, such as *Bacteroides massiliensis, Bacteroides dorei, Bacteroides thetaiotaomicron*, and *Bacteroides ovatus* were inversely associated with fecal SARS-CoV-2 load. It is worth noting that all these species are known to be associated with downregulation of ACE2 expression in murine colon, suggesting that these bacterial species could be beneficial to patients by reducing SARS-CoV-2 entry into target cells. In contrast, the *Firmicutes* species *Erysipelotrichaceae bacterium* showed positive correlation with fecal SARS-CoV-2 load, suggesting that this bacterial species could increase intestinal SARS-CoV-2 infection and replication. Fecal calprotectin, a biomarker of inflammatory response in the gut, was found elevated in COVID-19 patients with diarrhea (147). In a recent paper, the microbiota from COVID-19 patients was found to be characterized by an higher relative abundance of genera *Streptococcus, Veillonella, Fusobacterium, Clostridium, Lactobacillus* and *Bifidobacterium* whereas *Bacteroidetes, Roseburia, Faecalibacterium, Coprococcus, Parabacteroides*, and *Sutterella* (148). Another recent preprint (not peer reviewed) reported that based on proteomic data from 31 COVID-19 patients that identified biomarkers of unbalanced immune system (including IL-1β, IL-6, TNF-α, hsCRP), the screening of a cohort of 990 individuals without infection using the combination of fecal metabolomic analysis and machine learning model, found differences which could be indicative of the predisposition of individuals to inflammation and severe COVID-19 (15). The authors linked inflammation with high abundance of some genus, such as *Blautia* (positively associated with IL-10) and *Lactobacillus* (positively associated with IL-6 and IFN-γ).

**Figure 3:**
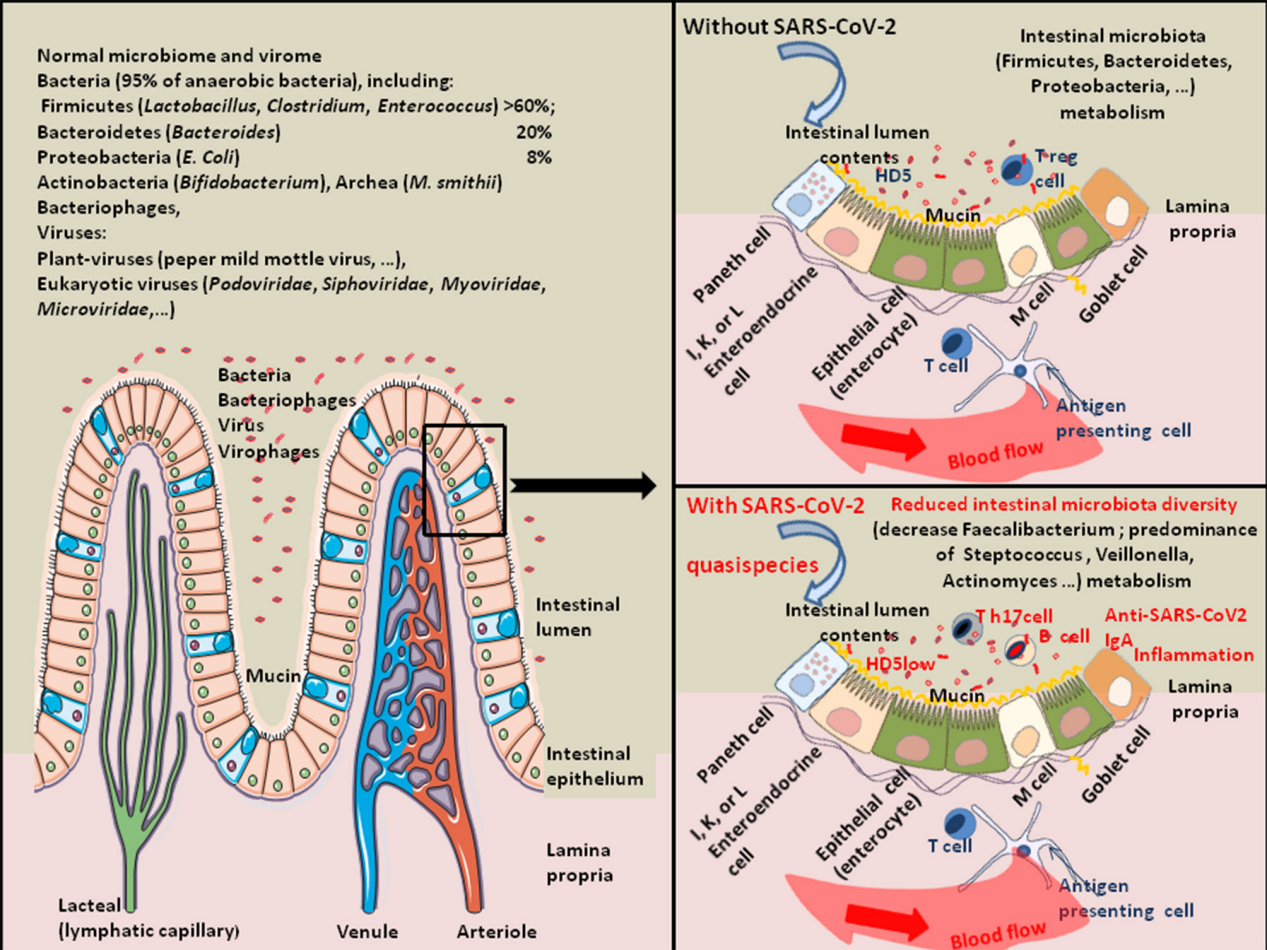
Schematic representation of SARS-CoV-2-associated intestinal dysbiosis. Once in the GIT, SARS-CoV-2 acts on the gut microbiota homeostasis and sometimes induces severe dysbiosis. About 100 trillion bacteria present in the intestinal lumen compose the human gut microbiota. It is a quite complex ecosystem, with over 1,000 bacterial species and 7,000 strains, in which the phyla Firmicutes (species, such as *Lactobacillus, Enterococcus*, and *Clostridium*) and Bacteroidetes (species, such as *Bacteroides*) account for the majority of species. Other phyla including Proteobacteria (*Escherichia coli*), Actinobacteria (Bifidobacteria), Cyanobacteria, Fusobacteria, and Verrucomicrobia are also present in lower abundance (149). The commensal intestinal microbiota is limited to the epithelium-distal mucus layer, while the epithelium-proximal mucus is largely devoid of bacteria. Viruses are also present in the human gut. They include bacteriophages, *Myoviridae, Siphoviridae, Podoviridae, Tectiviridae, Inoviridae, Microviridae*, among others (150). The gut microbiota expresses enzymes allowing the production of essential vitamins (such as vitamin K, B1, B6, B9, and B12). Bacteria from the Firmicutes, Bacteroidetes, and Actinobacteria phyla are involved in bile acids metabolism, liberating free primary bile acids, up-regulating the mucosal defenses and controlling the cholesterol homeostasis. Butyrogenic bacteria (such as Firmicutes), are ubiquitously present in the gut microbiota of healthy humans. These bacteria are very sensitive to oxidative stress. They produce butyrate, an end-product of anaerobic bacteria fermentation of non-digestible carbohydrates is considered a crucial protective molecule against inflammation. The butyrate inhibits the histone deacetylase HDAC, increases the junctional adhesion molecules JAM/occludin involved in the stability of tight junctions, and antagonizes the peroxisome proliferator-activated receptors (PPARs) that control inflammation [see the review (151) for details]. Many viruses (e.g., rotaviruses, caliciviruses, astroviruses, enteric adenoviruses, toroviruses, and parechoviruses) are known to induce gastroenteritis in humans (40). Coronaviruses are also frequently associated with diarrheal disease in humans. Regarding SARS-CoV-2, studies of gut microbiota have indicated a decrease in bacteria diversity in severe COVID-19 patients characterized by a decrease in Faecalibacterium (*Faecalibacterium prausnitzii*) and predominance of *Streptococcus, Veillonella, Actinomyces, Clostridium, Bacteroides*. The expression of antimicrobial compound HD5 and Vitamin D3 were also found reduced in COVID-19 patients.

These results support the hypothesis that SARS-CoV-2 infection is associated with a reduced production of antimicrobial agents, a reduced bacterial diversity (e.g., loss of beneficial bacteria) and a higher relative abundance of opportunistic pathogens (138, 139, 146, 148). This dysbiosis can be at the origin of the inflammation, tissue damage, and physical intestinal barrier loss associated with secondary bacterial lung infection (Figure 3). These alterations in microbiota diversity are likely to increase the risk to severe COVID-19 and disease progression.

## Modulation of Butyrate, Tryptophan, and Vitamin D3 Levels in COVID-19

**Figure 4:**
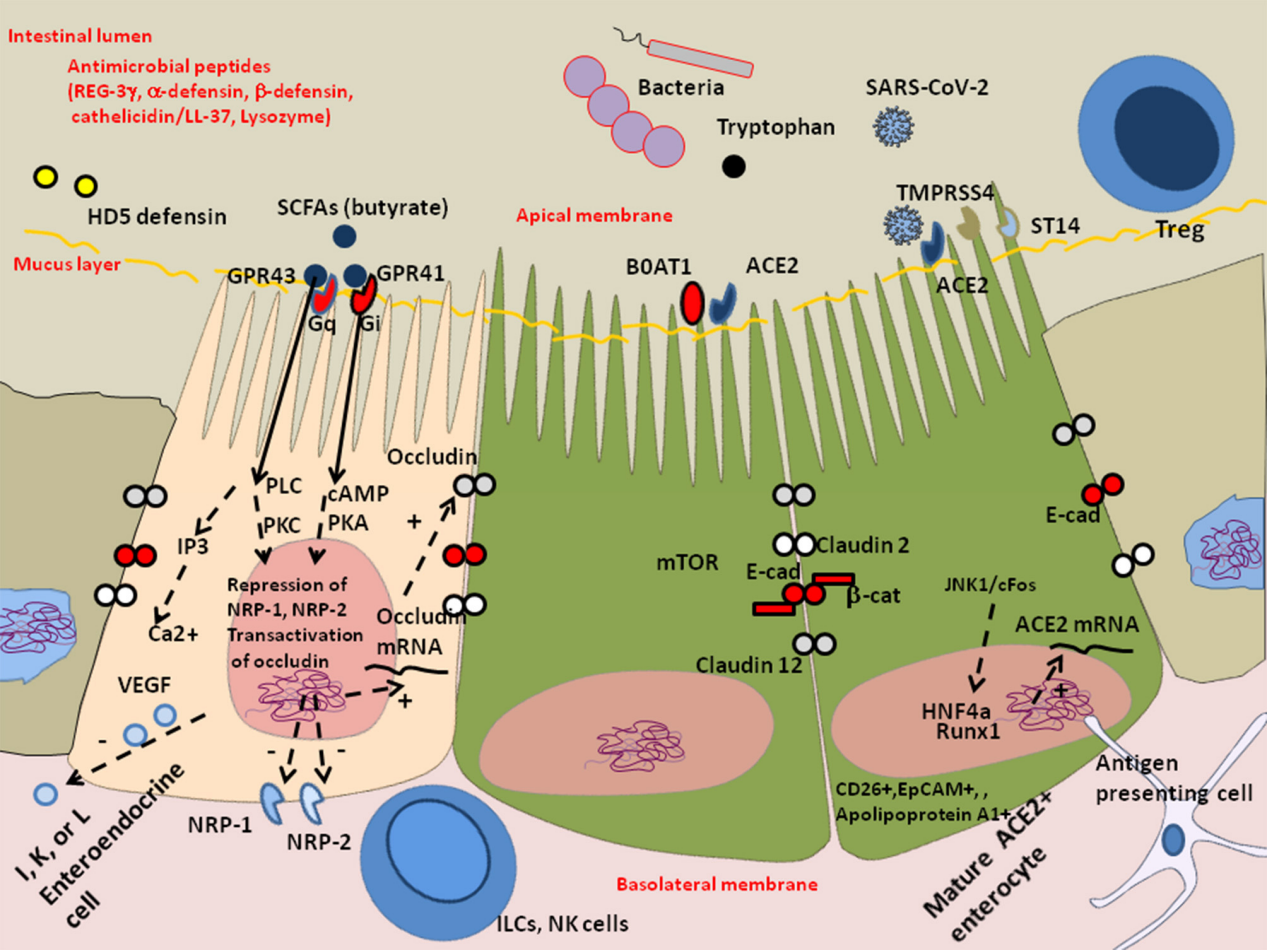
Schematic representation of the protective effect of butyrate against dysbiosis and its proinflammatory effects. The enteroendocrines I, K, and/or L epithelial cells express heterotrimeric guanine nucleoside-binding protein (G-protein) coupled cell surface receptors (e.g., FFA2/GPR43 and FFA3/GPR41) that bind the short-chain fatty acids (SCFAs) and trigger signal leading to the regulation of the glucose homeostasis and the secretion of hormones influencing appetite. These cells also express the NRP-1 expected to enhance SARS-CoV-2 infectivity and produce the vascular endothelial growth factor (VEGF) involved in the permeability of the capillary system. In COVID-19 patients, the decreased microbiota diversity probably influence the production of SCFAs acting on enteroendocrine cells through G-protein-coupled receptor that secrete bioactive compounds. In particular, the absence of cross-feeding bacteria that help maintain butyrate production by butyric acid bacteria can aggravate intestinal inflammation, tissue damage, translocation of toxins and pathogens. Besides being a major source of energy allowing cells to escape autophagy, butyrate acts on the epigenetic regulation of genes by inhibiting histone deacetylase (HDAC), it represses NRP-1 and NRP-2 expression and increases expression of junctional adhesion molecules occludin. Symbols: (+) means activation; (–) means: inhibition.

Butyrate, considered a protective molecule against inflammation is the end-product of anaerobic bacteria fermentation of non-digestible carbohydrates and also a component of dairy products (e.g., butter, milk, and cheese). By a mechanism of cross feeding, the intestinal symbiotic microbiota contributes to maintain the production of butyrate by butyric acid bacteria ubiquitously present in the gut microbiota of healthy humans. These bacteria also participate in the inhibition of pathogens growth by competing for nutrients and prevent toxin translocation by maintaining the integrity of the intestinal epithelium. Their mode of action is to metabolize carbohydrates to obtain short-chain fatty acids (SCFAs) including acetate, propionate, and butyrate (152). Yet, they are highly sensitive to oxidative stress. The abundance of *Faecalibacterium prauznitzii*, which is able to use acetate as a source for butyrate production, is significantly decreased in COVID-19 patients, although it is one of the most abundant Firmicutes in the gut of healthy humans (41, 151). It can therefore be hypothesized that butyrate is low in COVID-19 patients and insufficient to trigger secretion of bioactive compounds from enteroendocrine cells of the gut expressing the butyrate heterotrimeric guanine nucleoside-binding protein-coupled receptors GPR41 and GPR43 (153), which contributes to worsening dysbiosis. Butyrate could be added to the diet of the patients to counter the loss of obligate cross-feeding bacteria contributing to homeostasis (154). Butyrate was found to downregulate NRP-1 and VEGF in colorectal cancer cell lines and fecal butyrate levels are inversely proportional to NRP-1expression *in vivo* (135, 155). Conversely, the reduction of butyrate allows the expression of NRP-1, which likely contributes to SARS-CoV-2 infectivity of the GIT through binding to furin-cleaved substrates in the viral spike (130) (Figure 4). The use of butyrate as a supportive treatment for COVID-19 has already been proposed (156). High intestinal lumen butyrate interacts with both GPR41 and GPR43. Its binding to GPR43 activates the G-proteins which stimulates phospholipase C (PLC) leading to generation of diacyglycerol (DAG) which activates protein kinase C (PKC) and, inositol triphosphate which triggers Ca^2+^ release from the intracellular stores. Its binding to GPGR41 activates proteine kinase A (PKA) (157). Therefore, butyrate supplementation to restore high intestinal butyrate levels could possibly reduce infectivity of intestinal epithelial cells with SARS-CoV-2 and prevent autophagy.

**Figure 5:**
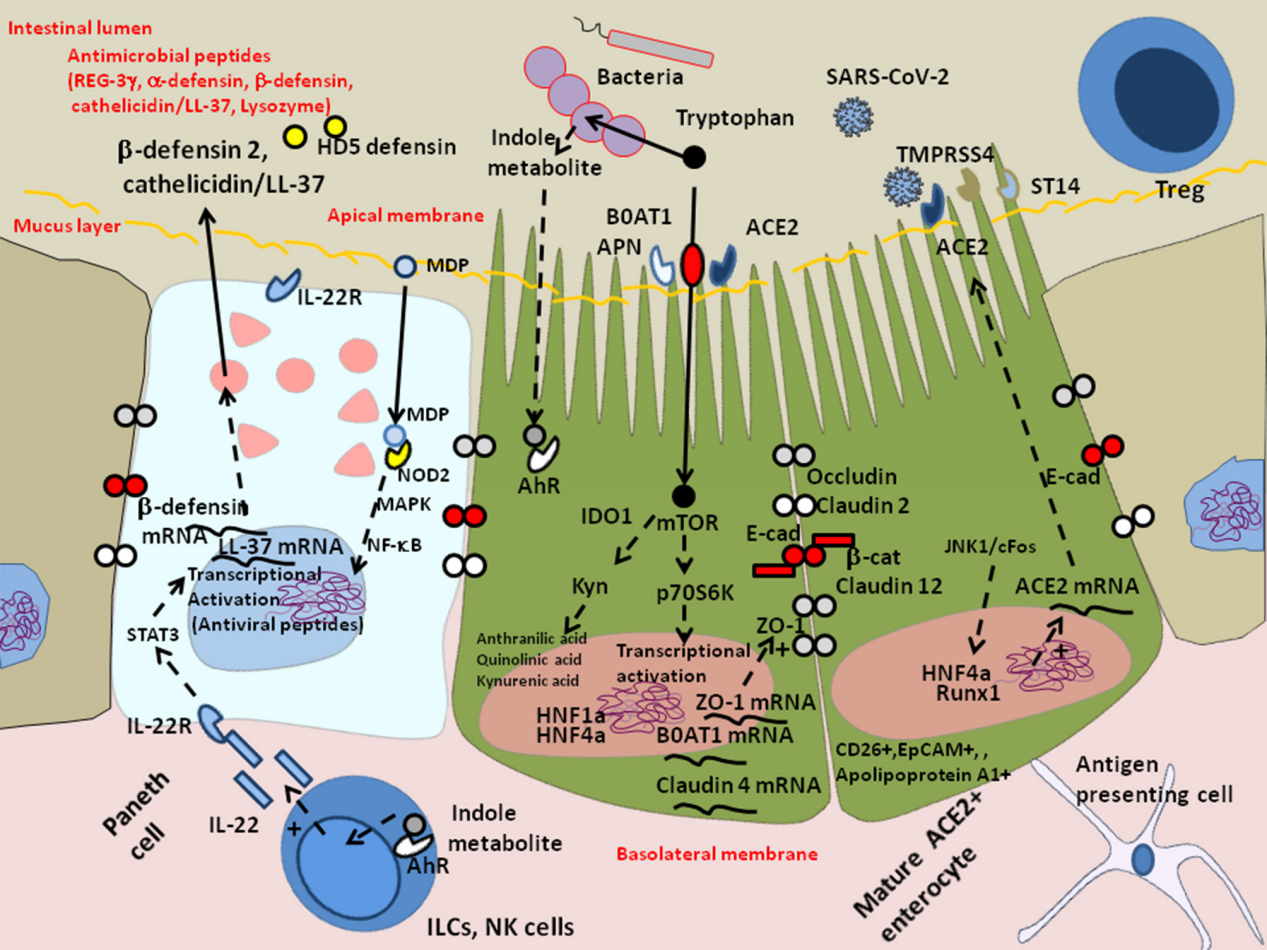
Interaction between the Tryptophan receptor channel B^0^AT1 and ACE2 at the surface of enterocytes. The main role of enterocytes is to ensure the absorption of nutriments. ACE2, expressed by enterocytes is necessary for the surface expression of the amino acid transporter B^0^AT1 in the intestinal epithelium. Tryptophan (Trp) is an essential amino acid obtained from food and whose assimilation depends on B^0^AT1. It mediates crosstalk between the intestinal mucosal immune system and the microbiota. Trp directly activates the mammalian target of rapamycin (mTOR) pathway and enhances tight junctions though increased expression of cell adhesion molecules Zonula occludens (ZO-1, ZO-2) and likely E-Cad. In addition, Trp promotes the IL-22/IL-22R-mediated expression of endogenous AMPs, such as β-defensin and LL-37 by Paneth cells, which in turn influence the composition of the intestinal microbiota. It is worth noting that HD5 binds ACE2. It can be hypothesized that following infection with SARS-CoV-2, tryptophan cannot get properly absorbed due to the reduced expression or the absence of ACE2/ B^0^AT1, leading to aberrant secretions of AMPs, and altered microbiota (decreased microbiota diversity), which confers susceptibility to intestinal inflammation. Symbols: (+) means activation; (–) means: inhibition.

As already discussed in this minireview, ACE2 is required for expression of the neutral amino acid transporter B^0^AT1 (158). Steric hindrance to the B^0^AT1 binding site on ACE2 or down-regulation of ACE2 due to the presence of SARS-CoV-2 is likely to display impairment in tryptophan uptake. In homeostatic condition, tryptophan is used by the host indoleamine 2,3-dioxygenase (IDO)_1_ to be converted to Kynurenine, and IDO_1_ exerts its biological effects mainly through the generation of downstream metabolites that suppress effector T-cell function, and favor the differentiation of regulatory T cells (Treg) (159). Several indole metabolites including indole, indole propionic acid, indole acetic acid, and tryptamine are produced by metabolism of tryptophan through the gut microbiota indole pathway that involves commensal species, such as *Peptostreptococcus russellii, Lactobacillus* spp., and *Clostridium sporogenes* (160). These indole metabolites have been described as activators of the aryl hydrocarbon receptor (AhR) (161). AhR promotes IL-22 production from innate immune cells (ILCs), natural killer T (NKT) cells, CD4^+^ lymphocytes cells, which stimulates the IL-22 receptor on intestinal epithelial cells triggering Stat3 activation and the induction of mucosal defense, mucin production by Goblet cells and the induction of AMPs release by Paneth cells (105, 162) (Figure 5). A recent observation was reported indicating that COVID-19 infection results in alterations of the kynurenine pathway and fatty acid metabolism that correlate with IL-6 serum levels (163), which is consistent with impairment of tryptophan metabolism leading to synthesis of N-formyl-L-kynurenine, L-kynurenine, and anthranilic acid through the IDO_1_/Tryptophan 2,3-dioxygenase (TDO) pathway (164). Indeed, impaired tryptophan uptake leads to aberrant mammalian target of rapamycin (mTOR) protein kinase and p70^S6kinase^ activation and lower production of antimicrobial peptides (AMPs) from enterocytes (106) and Paneth cells granules, with a reduced production of lysozyme, RegIIIγ, cystein-rich cationic peptides with antibiotic and antiviral activity (e.g., α-defensin HD5 and HD6), leading to a change in the composition of the microbiota and an increase in bacterial translocation in situations of loss of the intestinal physical barrier. It was also reported that mice deficient in ACE2 have altered microbiota and increased susceptibility to intestinal inflammation induced by epithelial damage. The transplantation of their microbiota into germ-free mice increased the propensity of recipient mice to develop severe colitis which can be prevented by dietary amino acid tryptophan (95). This suggests that the addition of tryptophan to the diet of COVID-19 patients suffering from diarrhea may improve their health (160).

**Figure 6:**
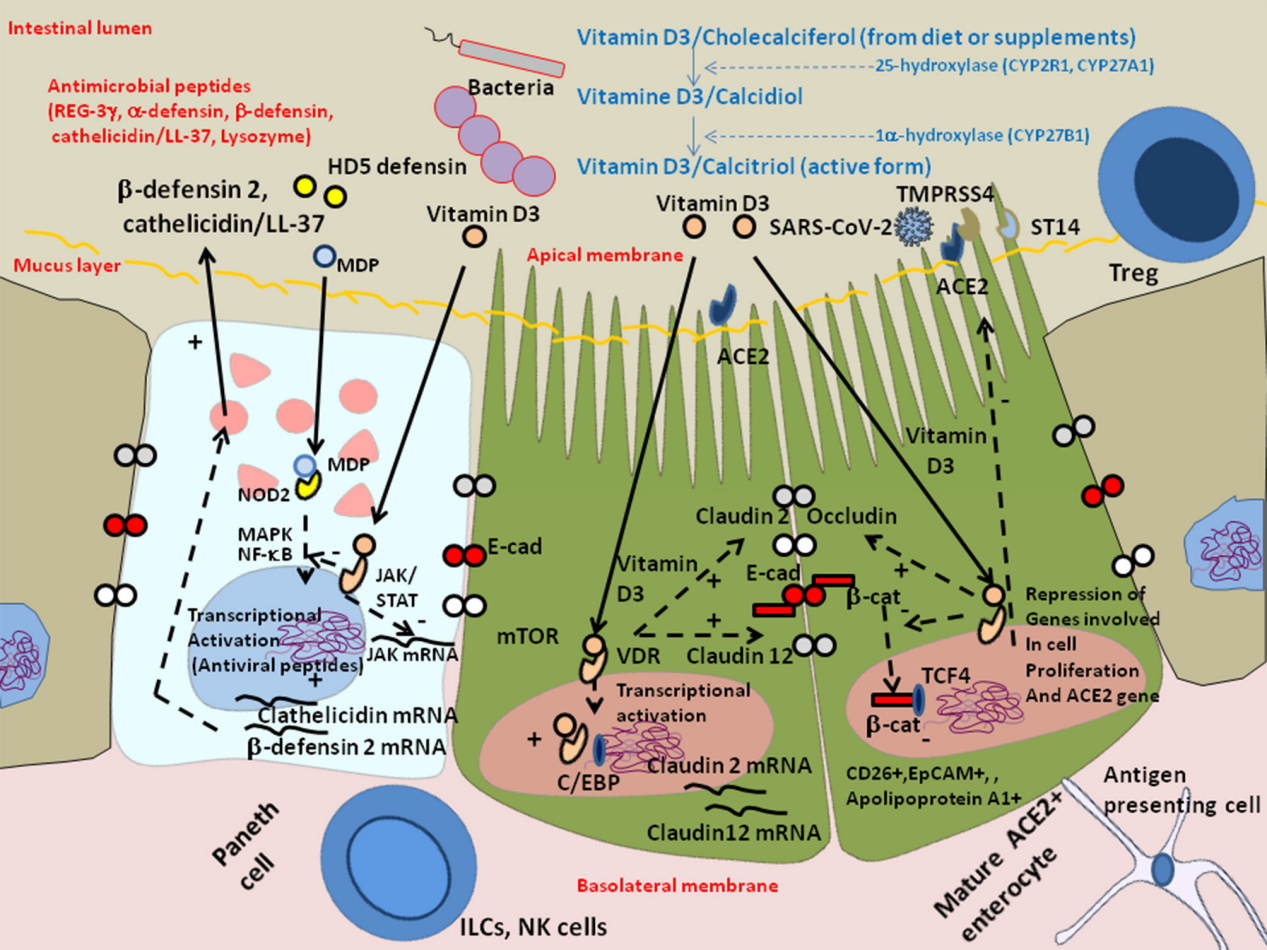
Schematic representation of the protective effect of Vitamin D against dysbiosis and its proinflammatory effects. Vitamin D/cholecalciferol from diet or supplements is hydroxylated to 25-hydroxy-vitamin D or calcidiol by the cytochrome P450 hydorolases CYP2R1 and CYP27A1, then it is hydroxylated at the 1 alpha position by CYP27B1 to generate calcitriol which is the metabolically active form of vitamin D3. Vitamin D receptor (VDR) maintains the Paneth cell alertness to pathogens in intestinal disorders. The Paneth cells produce antimicrobial proteins (e.g., C-type lectin REG3γ, α-defensins, β-defensins, cathelicidins, and lysozyme) in response to infection. Genetically and environmentally regulated VDR in the Paneth cells may set the threshold for the development of chronic inflammation. Vitamin D3/VDR upregulates cathelicin/LL-37 and β-defensin 2 and downregulates the JAK/STAT and NF-kB pathways. In the presence of Vitamin D3, the VDR expressed in enterocytes translocates to the nucleus where it cooperates with the C/EBP transcription factor to increase Claudin 2 and Claudin 12 gene transcription, increasing the pools of cytoplasmic Claudins, two molecules that contribute, together with E-cadherin (E-Cad), to the integrity of epithelial tight junctions. The activation of VDR by vitamin D3 also suppresses the cytoplasmic release of β-catenin from E-cad thus decreasing the levels of nuclear β-catenin and reducing the levels of β-catenin/T cell factor (TCF) complexes that are required for the expression of genes involved in cell proliferation (such as cyclin D). Symbols: (+) means activation; (–) means: inhibition.

Recently it was reported that among 12 patients with respiratory distress, 11 (91.7%) had one or more nutrient deficiencies, with vitamin D deficiency being observed in 76% of COVID-19 patients vs. 43.3% of controls (165, 166). Vitamin D is provided by the food bolus (e.g., fatty fish, olive oil, calf liver, chocolate). The vitamin D receptor (VDR), a nuclear receptor expressed in intestinal enterocytes of the proximal colon and particularly Paneth cells (it is also distributed in a large variety of cells, such as bronchial epithelial cells, lymphocytes, monocytes, skin keratinocytes, and distal renal cells) (167, 168), is an important contributor to the intestinal homeostasis. Vitamin D deficiency is common in patients with inflammatory bowel disease of the GIT (169), cystic fibrosis and chronic obstructive pulmonary disease in lung (170). It was reported that old men with the highest levels of the active form of Vitamin D (1α,25-dihydroxyvitamin D) are more likely to possess butyrate-producing *Firmicutes* and *Clostridia* bateria (171). The 1α,25-dihydroxyvitamin D_3_ (calcitriol calcemic hormone) up-regulates cathelicidin (the anti-microbial peptides LL-37) and β-defensin 2 (172). The JAK/STAT3 pathway is over-activated in response to intestinal dysbiosis and VDR transcriptionally regulates Jak2 to maintain homeostasis (173). Using ACE2 as bait to build a genomic-guided molecular map of upstream regulatory element it was found that JNK1/cFos, HNF4α, Runx1 are activators of ACE2 gene expression while VDR (activated by HNF4α), is a repressor of ACE2 (174) (Figure 6). Vitamin D supplementation protects the intestinal epithelium against bacterial infection and invasion by acting on the bacterial induced activation of the NF-κB pathway. Vitamin D triggers the interaction between VDR and the p65 subunit of NF-κB, reducing its phosphorylation and nuclear translocation (175). This leads to a reduction in intestinal epithelial apoptosis, maintenance of the integrity of the intestinal mucosal barrier (176, 177). Moreover; it may increase the levels of Treg lymphocytes (known to participate in the control of inflammation), which have been reported to be low in many COVID-19 patients (178) and attenuate Th1 and Th17 responses (177). VDR physically interacts with β-catenin and regulates the E-Cad expression involved in epithelial junctions through repression of β-catenin (179). Activation of VDR by vitamin D induces expression of CYP3A, a cytochrome P450 enzyme that detoxifies the secondary bile acid lithocholic acid (LCA), in the intestine (180). Moreover, vitamin D decreases rhinovirus replication and increase interferon and anti-microbial peptide cathelicidin/LL-37 which demonstrates antiviral activity against respiratory enveloped viruses, such as influenza and respiratory syncytial virus (RSV) (181–184). An increased mortality (21 vs. 3.1%) was reported in vitamin D-deficient COVID-19 patients (185). Indeed, insufficient vitamin D levels increased hospitalization and mortality from COVID-19 (186). A preliminary study on residents of a nursing-home who received chronic vitamin D supplementation with regular maintenance boluses (single oral dose of 80,000 IU vitamin D3 every 2–3 months), suggests that regular vitamin D3 intake halves the risk of fatal outcome of COVID-19 (187).

## Discussion

Respiratory and gastrointestinal epithelia share a common embryonic origin in the primitive foregut which likely account for shared functional characteristics (188). Although SARS-CoV-2 was first described as a virus capable to infect pneumocytes, we highlight here the possibility of gastrointestinal system as a potential target for enterocyte-tropic or dual-tropic SARS-CoV-2.

It is currently unclear whether SARS-CoV-2 can be transmitted through the fecal-oral route (GIT being considered in that case a primary site of infection), if the upper GIT may be involved in SARS-CoV-2 entry followed by replication in the intestinal epithelium prior to dissemination to other tissues, or if the virus can spread from a primary pulmonary site of infection into the gastrointestinal system (secondary site). It is also unclear whether or not SARS-CoV-2 quasispecies contain viruses with a preferential lung tropism and other with preferential intestinal tropism, or if some SARS-CoV-2 are dual tropic viruses. In addition to the isolation and sequencing of the SARS-CoV-2 from the upper respiratory tract there is an imperative necessity to collect stool samples from COVID-19 patients to isolate the SARS-CoV-2 spreading in the GIT and to compare their genome and biological properties with those of SARS-CoV-2 isolated from the respiratory tract. A high virus titer in the stool might indicate a higher risk of transmission via feces.

Elegant work has shown that SARS-CoV-2 uses the ACE2 receptor for binding and the serine protease TMPRSS2 for the S glycoprotein priming, and demonstrated that the serine protease inhibitor camostat mesylate which is active against TMPRSS2, partially blocked SARS-CoV-2 spike driven entry in the human Caco2 intestinal (ACE2^+^, TMPRSS2^+^) cells (189, 190). It was recently reported that when the Caco2 cells were exposed to vesicular stomatitis virus (VSV) particles pseudotyped with chimeric spike from SARS-CoV-2 that carry receptor binding domain (RBD) variant sequences from different betacoronaviruses, appropriate RBD sequence is required for infection whereas most RBD are incompatible with infection (21). When Caco2 cells were exposed to SARS-CoV-2 S pseudovirions (94), adhesion was observable by confocal microscopy after 1 h of incubation. So far, the infection and replication of a laboratory strain of SARS-CoV-2 at a multiplicity of infection (MOI) of 0.1 for 2 h at 37°C in human Caco2 (intestinal) cells and Calu3 (pulmonary) cells were reported to be comparable over a period of 120 h (191). It is worth noting that SARS-CoV-1 was shown to infect polarized Calu3 cells at the apical membrane and is also released at the apical membrane with evidence of cytopathic effect (CPE), whereas no CPE was reported during replication of SARS-CoV-1 on Caco2 cells (192). Preliminary data reported in a preprint (not peer-reviewed) available on the web site of our Institute, the IHU Méditerranée Infection (193), indicate that in Caco2 cells exposed to SARS-CoV-2 IHUMI2 grown in VERO-6 cells, the viral replication occur (according to RT-PCR monitoring) but no CPE was observed during the 7 days of cell culture. Other data reported in a preprint (not peer-reviewed) monitored Caco2 cellular toxicity 48 h following exposure to SARS-CoV-2 (MOI: 0.01) isolated in Germany from travelers returning from Wuhan (194). Caco2 cells infected with SARS-CoV-2 produce filopodia protrusions extending out from the cell surface containing viral particles (195).

ACE2 modulates innate immunity and influences the composition of the gut microbiota diversity which can explain GIT symptoms. Usually, the mucus layer present at the surface of the intestinal epithelium, the antimicrobial peptides produced by Paneths cells and other epithelial cells of the intestine, the commensal intestine microbiota competing with possible infectious pathogens, are acting as first line of intestinal innate defense while the homotypic interaction of E-cad in trans acts as a second defense line protecting the host against intruder transmigration (137). It is likely that during SARS-CoV-2 infection, infected enterocytes died from virus-induced apoptosis or autophagy leading to viral clearance through dead enterocytes renewal. Regarding innate immunity, SARS-CoV-2 infection of enterocytes was found to induce a strong IFN response and the production of cytokines (e.g., the IFNγ-inducible cytokine CXCL10 known to bind CXCR3 receptor and to induce inflammation) (97), similar to that observed during infection of respiratory tissues (196). Recently, it was reported that GIT infection by SARS-CoV-2 was associated with a significant reduction in COVID-19 severity and mortality with an accompanying reduction in key inflammatory proteins including IL-6, CXCL8, IL-17A, and CCL28 (197). It was reported that *Lactobacillaceae* fermentation produce bioactive peptides with the capability to inhibit ACE (198, 199) likely reducing the concentration of angiotensin II that is responsible for proinflammatory signals in COVID-19 patients (4). These peptides could possibly bind to ACE2 since the active site of ACE2 contains a zinc-metallopeptidase motif and share 42% sequence homology with the amino-terminal domain of ACE (200), and prevent ACE2 interaction with SARS-CoV-2. The recent investigation of COVID-19 patients microbiota provided evidence of dysbiosis with a significantly reduced bacterial diversity (including a decreased abundance of *Faecalibacterium prausnitzii* known to prevent inflammation; this bacterium can use acetate as a source for butyrate production) and higher relative abundance of opportunistic pathogens (*Streptococcus, Rothia, Veillonella*, and *Actinomyces*) which can aggravate inflammation (139, 146, 148). This is likely associated with damage of epithelial tight with cleavage of E-cadherin and release of soluble E-cadherin as previously described (137), a phenomenon also observed during chronic obstructive pulmonary disease (141). Epithelial breakdown allows the establishment of invasive bacterial infections possibly resulting in secondary bacterial lung infection.

Lung dysfunction as a result of inflammatory bowel disease was reported more than 40 years ago (201). Since then, increasing evidence supports the idea that alteration in the gut microbial species can alter the inflammatory state and the immune response and, ultimately, influence disease outcome in the lungs (138, 139, 202). For example, in Influenza A virus infection, a change in lung microbiota composition with enrichment in *Streptococcus* and decreased abundance in *Pseudomonas* has been reported (203) as well as a shift from *Bacillus* to *Lactobacillus* in the lung microbiota with concomitant reduction of bacterial species diversity for the gut microbiota (204). In a murine model, it was observed that the reduction of the gut microbiota diversity by antibiotics increased the susceptibility to Influenza virus in the lung (205). Moreover, an increased abundance of *Streptococcus* and *Staphylococcus* was reported in the bronchoalveolar lavage fluid of mice inoculated intra-nasally with H1N1 (206). Within rhinovirus-infected patients diagnosed with chronic obstructive pulmonary fibrosis, an increased abundance of *Haemophilus influenzae* was observed compared to controls (207). Dickson et al. reported that gut associated species were present in higher abundance in the lungs of patients with acute respiratory distress syndrome (ARDS) than in healthy controls (208). Similarly, enrichment of lung microbiota with bacteria found in the GIT is correlated with the onset of acute respiratory distress syndrome and severity of COVID-19 (209, 210). In the lung tissue of deceased patients with COVID-19 the most prevalent genera were *Acinetobacter* (80.7%), *Chryseobacterium* (2.7%), and *Burkholderia* (2.0%) (211). The assumption can also be made that lung microbiota changes can signal to the gut and might contribute or amplify systemic inflammation and gastrointestinal disorders as observed for other viral infection (212). For example, influenza-induced IFN produced in lung promotes depletion of obligate anaerobic bacteria and enrichment of Enterobacteriaceae in the GIT and leads to a proinflammatory gut environment (213).

The fact that SARS-CoV-2 infection of enterocytes leads to decreased production of antimicrobial peptides may also have indirect adverse effects on distant tissues (e.g., heart, lungs), since the antimicrobial PR-39 peptide has been shown to provide cardioprotection by preventing leukocyte adhesion and emigration (214). However, the model of antimicrobial peptides that provide cardiovascular protection is not as simple, since other antimicrobial peptides have the opposite effect (e.g., α-defensins have been linked to atherosclerosis and the antimicrobial peptide LL-37 is highly expressed in atherosclerotic plaques) (112, 113, 215). Once GIT epithelium is damaged in COVID-19 patients, the interaction between the virus and NRP-1 expressed on cells of the crypts of colonic epithelium these cells are likely to trigger the release of VEGF-containing granules from enteroendocrine cells, followed by a modulation of the permeability of the capillary system. It is known that microvascular injury and obstructive thrombo-inflammatory syndrome represent the primary causes of COVID-19 lethality (216, 217).

We can hypothesize that in COVID-19, the gastrointestinal dysbiosis is the consequence of a cascade of events that are found in most of the pathological processes, namely a loss of bacterial diversity, in particular of “beneficial” bacteria, a greater abundance of “harmful” bacteria associated with damage to the epithelium. This dysbiosis is followed by the induction of a pro-inflammatory response that results in an immunological shift from Treg cells to Th1 and Th17 cells. Maintaining a balanced immune response in COVID-19 appears to be essential to improve patient outcome. Therefore, in order to reduce the intestinal proinflammatory states in COVID-19 patients, one strategy could be to promote butyrate (4 g sodium butyrate daily), L-tryptophan (4 mg/Kg of body weight daily) and Vitamin D3 (5,000–10,000 IU daily) supplementation to the patients diet in addition to a well-chosen antibiotic therapy and anti-inflammatory molecules. Controlled trials should be conducted to evaluate this therapeutic strategy.

## Author Contributions

CD, J-CL, and DR contributed to conceive the manuscript. CD wrote the paper. DR obtained the funding for this study. All authors reviewed and approved the final version of the manuscript.

## Conflict of Interest

The authors declare that the research was conducted in the absence of any commercial or financial relationships that could be construed as a potential conflict of interest.
